# Geographic variation in bacterial assemblages on cane toad skin is influenced more by local environments than by evolved changes in host traits

**DOI:** 10.1242/bio.059641

**Published:** 2023-02-06

**Authors:** Chava L. Weitzman, Mirjam Kaestli, Alea Rose, Cameron M. Hudson, Karen Gibb, Gregory P. Brown, Richard Shine, Keith Christian

**Affiliations:** ^1^ Research Institute for the Environment and Livelihoods, Charles Darwin University, Darwin, NT 0909, Australia; ^2^ Department of Aquatic Ecology, Eawag, Swiss Federal Institute of Aquatic Science and Technology, 8600 Dübendorf, Switzerland; ^3^ School of Natural Sciences, Macquarie University, Sydney, NSW 2109, Australia

**Keywords:** *Bufo marinus*, Invasive species, Rapid evolution, Skin bacteria

## Abstract

Bacterial assemblages on amphibian skin may play an important role in protecting hosts against infection. In hosts that occur over a range of environments, geographic variation in composition of bacterial assemblages might be due to direct effects of local factors and/or to evolved characteristics of the host. Invasive cane toads (*Rhinella marina*) are an ideal candidate to evaluate environmental and genetic mechanisms, because toads have evolved major shifts in physiology, morphology, and behavior during their brief history in Australia. We used samples from free-ranging toads to quantify site-level differences in bacterial assemblages and a common-garden experiment to see if those differences disappeared when toads were raised under standardised conditions at one site. The large differences in bacterial communities on toads from different regions were not seen in offspring raised in a common environment. Relaxing bacterial clustering to operational taxonomic units in place of amplicon sequence variants likewise revealed high similarity among bacterial assemblages on toads in the common-garden study, and with free-ranging toads captured nearby. Thus, the marked geographic divergence in bacterial assemblages on wild-caught cane toads across their Australian invasion appears to result primarily from local environmental effects rather than evolved shifts in the host.

## Teaser

**Summary:** Although multiple phenotypic traits have evolved in cane toads across their invaded range in Australia, divergences among toads in skin bacterial communities are more affected by local environments than by evolved shifts in the hosts.

## INTRODUCTION

A wealth of microbes inhabit all animal surfaces (Adair and Douglas, 2017). On amphibian skin, microbial communities are broadly characteristic for each host species; within a given habitat the microbiota is more similar among conspecific individuals than with heterospecifics (Christian et al., 2018; McKenzie et al., 2012; Walke et al., 2014). Even closely related hosts that occupy similar niche space in the same habitat harbour distinct communities (Christian et al., 2018). Host phylogeny broadly affects microbiome composition among amphibians (Ellison et al., 2019), while on a finer scale, host genotype impacts skin community composition within a host species (Belasen et al., 2021), giving rise to these species- and population-level patterns. Bacterial assemblages on amphibians consequently comprise rare taxa filtered from the host's environment by properties of the host's skin mucus (Walke et al., 2014). The microbial assemblage is determined by a balance between skin properties that select for specific bacteria and environmental factors that lead to variability among individuals. However, the relative influence of host-determined versus environmental factors on these symbiotic relationships is still largely unknown (Kohl, 2020).

Strong species-specific patterns in skin bacterial communities suggest that even widely separated populations retain a shared core microbial assemblage. Frogs can share core bacterial taxa with conspecific populations 40 km away (Christian et al., 2018; Hughey et al., 2017). However, geographic variation in environmental bacteria should broadly impact the bacteria available to colonise hosts. Consequently, although some specific bacterial taxa may be widely distributed on conspecific hosts, stronger core communities would be expected in higher-level bacterial groups [for example, amplicon sequence variants (ASVs) versus operational taxonomic units (OTUs) versus bacterial genera and families].

To distinguish the effects of host characteristics versus environmental factors in driving microbial composition, we need an experimental approach. For example, when Sierra Nevada yellow-legged frogs (*Rana sierrae*) in California were raised on water from the same sources, frogs from two source populations developed different skin bacterial assemblages (Jani and Briggs, 2018). This heritability in microbial assemblages indicates a role for host traits in determining skin microbial composition.

Invasive species offer excellent systems in which to examine rapid adaptation of hosts to divergent environments. Cane toads (*Rhinella marina*) have spread across much of tropical Australia in the past 90 years, stimulating rapid changes in toad morphology and behaviour in both heritable and phenotypically plastic ways (Rollins et al., 2015). For example, toads at the invasion front exhibit more exploratory behaviours, disperse farther, and have faster growth rates (Alford et al., 2009; Gruber et al., 2017; Phillips, 2009). Toads exhibit changes in epidermal thickness throughout the invasion and within drier environments (Kosmala et al., 2020), and in a common-garden setting, offspring from toads farther west in their Australian invaded range have increased capacity in some innate immune responses (Brown et al., 2015). As in other (non-invasive) anurans, skin microbial assemblages (an extension of the innate immune system) of cane toads in Australia exhibit geographic variation across populations, particularly between long-colonised versus recently colonised areas (Christian et al., 2018; Weitzman et al., 2019). Cane toads in Australia came from a single origin source with long-colonised sites genetically clustering with their Hawaiian source population (Selechnik et al., 2019), meaning that differences in skin microbiomes between populations have arisen independently during colonisation. Therefore, this species provides a good model not only for distinguishing between the effects of environment and host traits in determining the composition of microbial communities, but also providing a system in which both host traits and environmental characteristics have changed very recently.

In this study, we used a combination of bacterial microbiome sampling of wild toads from across northern Australia and a concurrent common-garden experiment at a single site to address predictions about microbial assemblages. The wild-toad samples were previously analysed to detect the potential for toad skin microbes to inhibit the fungal pathogen *Batrachochytrium dendrobatidis* (Weitzman et al., 2019). Here, we provide broader analyses of diversity from these wild sites to understand skin bacterial patterns on these invasive toads and to inform predictions of the hosts’ role in microbial assemblages.

First, we assessed differentiation of skin bacteria on toads from the four wild sites sampled. We expected to find geographical variation in assemblage composition, consistent with previous results in cane toads and other anurans (Christian et al., 2018; Hughey et al., 2017; Kueneman et al., 2014); but we also expected to find a group of core bacteria common to all wild populations. Next, we used the common-garden experiment to assess site-level impacts on skin microbe assemblages, which could occur through a combination of vertical (or pseudo-vertical) and horizontal transmission (Becker et al., 2014; McGrath-Blaser et al., 2021; Rebollar et al., 2016; Walke et al., 2011), and physiological/phenotypic differences among populations that affect microbial selection. We addressed this question by sampling not only the common-garden-toad offspring, but also their re-located parents. By comparing captive parents and their offspring with free-ranging toads collected at the same sites and from areas close to the location of the common-garden experiment, we could tease apart the relative influences of local environments and heritable host traits on microbial assemblages. A focus on heritable differences in microbiome assembly removes priority effects from the varied histories of wild-caught individuals. We expected to find both an ancestral population-level signal (reflecting host traits) and a unique signature in captive toads regardless of their geographic origin, due to conditions in captivity (Kueneman et al., 2022).

Because toads in differing environments likely encounter distinct microbes compared with those available to the toads held in our common-garden rearing facility, we analysed amplicon sequence variants (ASVs; unique sequences) as well as operational taxonomic units (OTUs; clustering sequences at 97% similarity). In comparing these two sequence-processing methods, we expected to find more similarity in OTUs than in ASVs between skin microbiomes of toads in the common-garden experiment, their re-located parents, and free-ranging toads.

## RESULTS

**Fig. 1.**
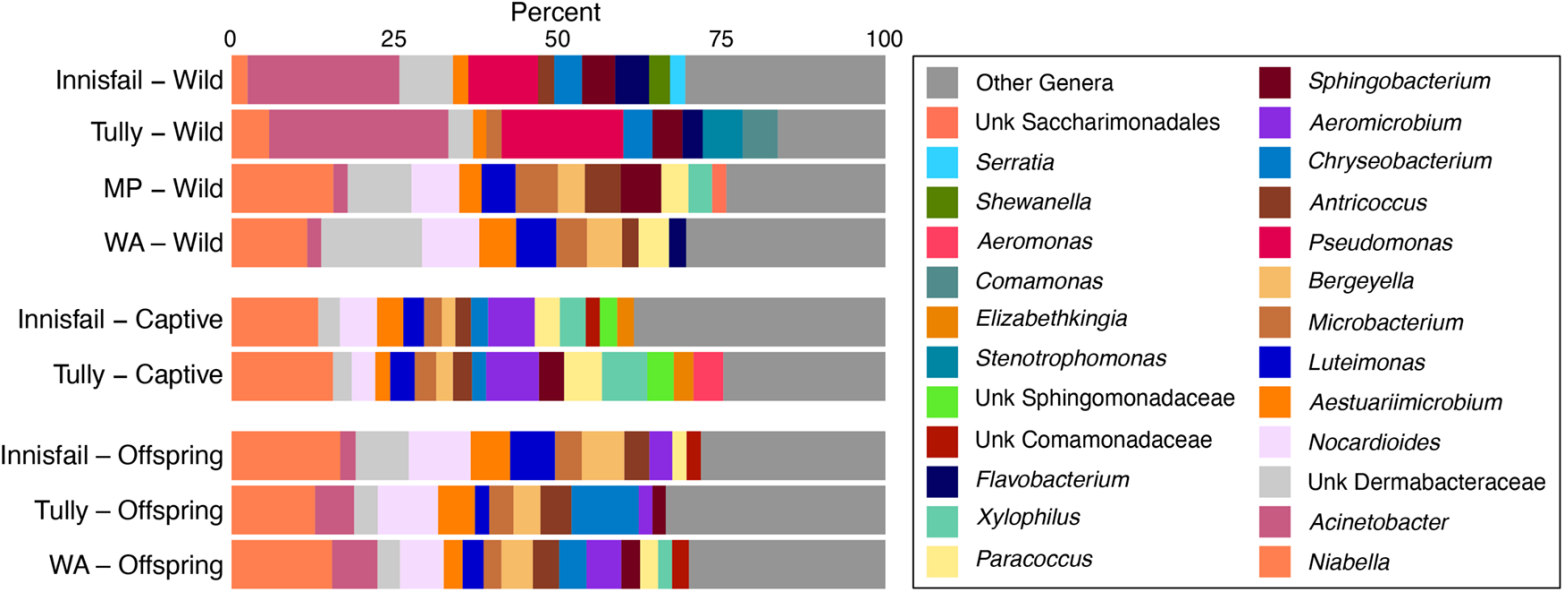
**Genera with relative abundance >2% per toad group from skin swab samples.** Values are averages within each toad group. Toad groups represent those present in the common-garden experiment offspring, relocated captive toads at Middle Point, and wild toads.

Common-garden toads were reared at Middle Point, Northern Territory, representing offspring of three sites from Queensland and Western Australia (Table 1). We collected skin swab samples from the common-garden offspring, the remaining captive parental toads, and wild toads from the ancestral sites and Middle Point to assess the impacts of environment and genotype on skin bacterial communities based on bacterial 16S rRNA amplicon sequencing. Sequencing resulted in 4,760,022 reads in 96 toad samples and controls. After filtering, we used 6025 bacterial ASVs and 3798 OTUs in 93 toad samples for analyses, with an average (±s.d.) of 28,206±13,660 bacterial reads per sample. Most reads were identified as Actinobacteriota, Proteobacteria, and Bacteroidota, with these three phyla constituting 82–100% of the reads per sample. Toads grouped by sampling site and ancestry had 11–16 genera representing at least 2% of the reads from the group, and these genera with high relative abundance accounted for approximately 70% of the reads per group (Fig. 1). Abundant genera included *Niabella*, *Acinetobacter*, and *Nocardioides*. Four ASVs were present in every sample, accounting for 2–49% of the reads per sample (four OTUs in every sample, at 2–50% of sample reads).

**Table 1.**
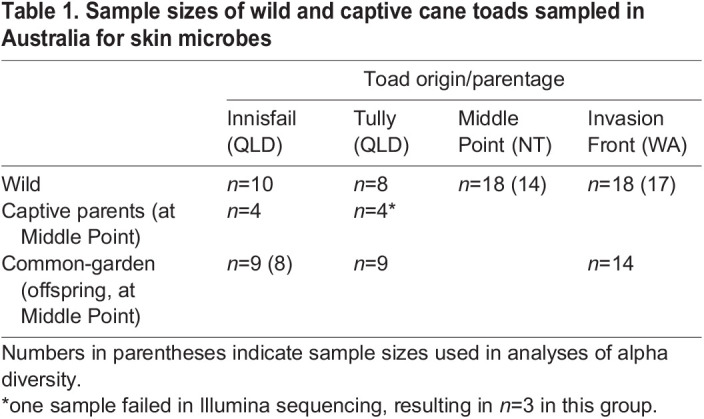
Sample sizes of wild and captive cane toads sampled in Australia for skin microbes

### Free-ranging toads

**Fig. 2.**
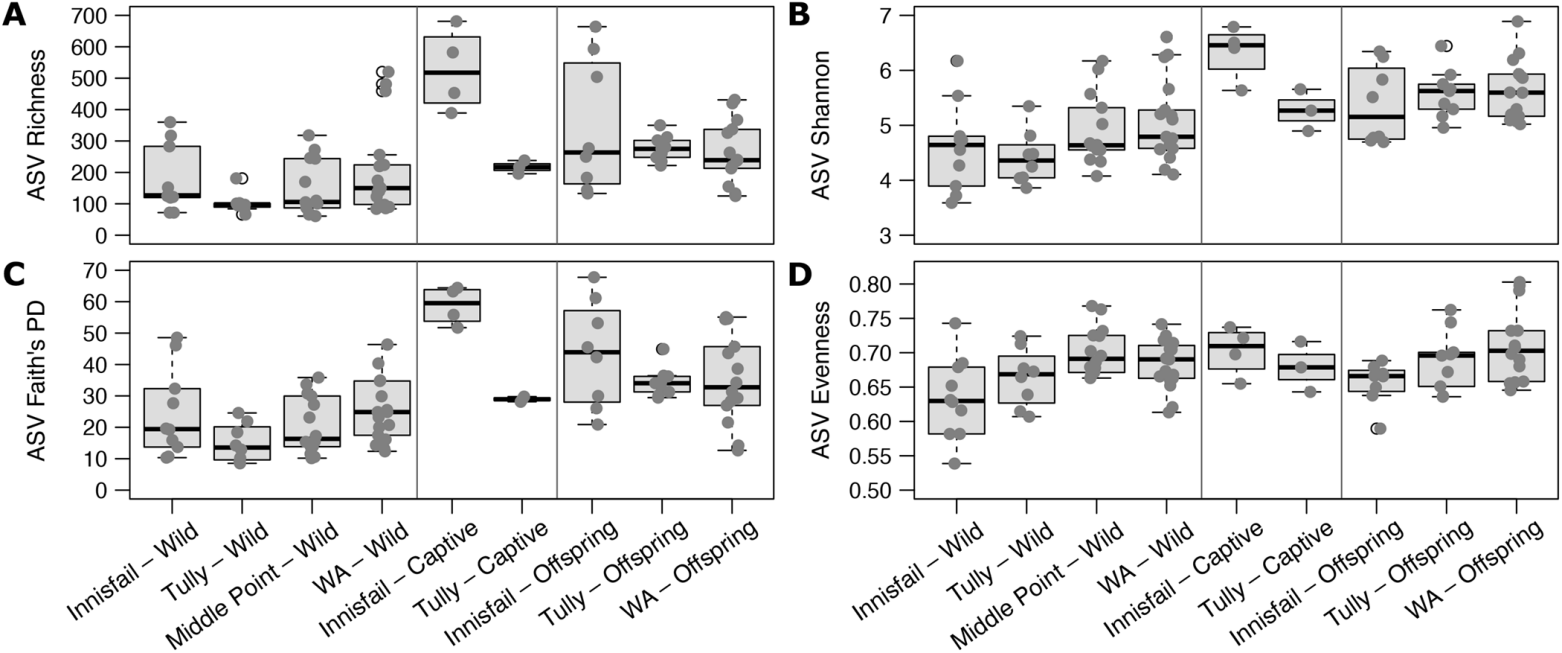
**Alpha diversity values in skin bacterial communities of captive and wild cane toads across Northern Australia.** Boxplots show the median, interquartile range, reasonable range of the data, and outliers (open circles). Vertical lines separate data between wild toads, captive relocated toads, and common-garden toad offspring.

**Fig. 3.**
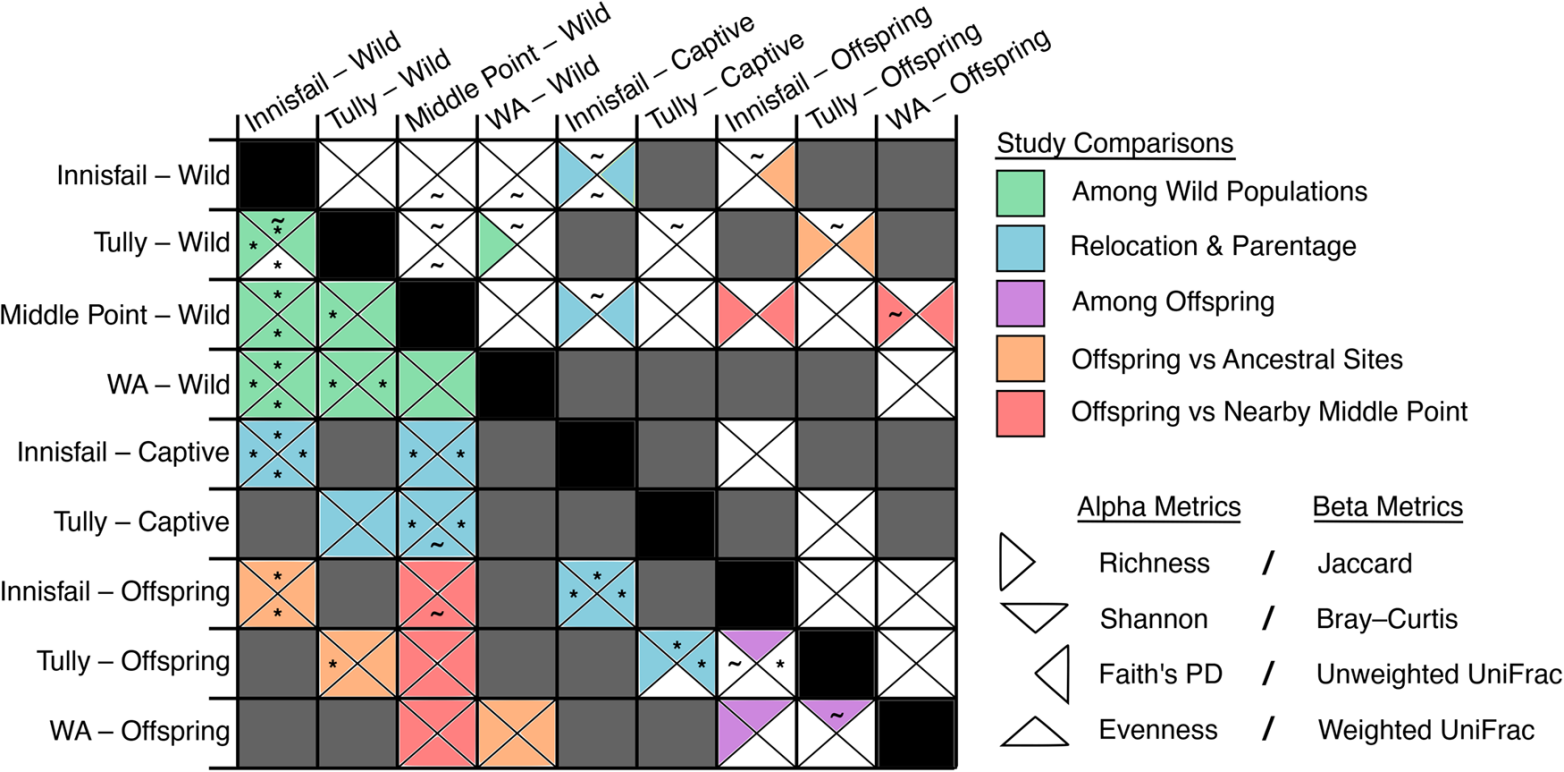
**Results of pairwise contrasts of diversity metrics (presented in Table 2) of bacterial ASVs on cane toad skin in Australia.** Results of alpha diversity analyses are above the diagonal, and beta diversity comparisons are below the diagonal. Sample sizes are provided in Table 1 and differ between alpha and beta diversity metrics. Dark-grey-shaded squares are pairwise comparisons not focused on in this study. Squares of comparisons of interest are separated into four quadrants, one for each diversity metric. Filled triangles indicate significant differences between groups, with color-coding by study question. Color-coding aids in identifying comparisons relevant for the questions of interest but is not necessary for interpretation. Significant pairwise differences between groups were rare for alpha diversity metrics (GLMs or LMs), but almost ubiquitous in beta diversity metrics (PERMANOVAs). Common-garden offspring of different ancestry had the most similarities in diversity metrics. ∼ indicates instances where the OTU comparison had an opposing significance (< or >0.05) to the ASV comparison. Asterisks denote pairwise differences in dispersion. All global analyses of dispersion were significant. WA: toads captured near the invasion front in Western Australia. This figure is available in table format in the supplementary information. Note that Shannon diversity and Pielou's evenness for ASV data did not have a significant interaction between origin site and captivity, so we do not report results of pairwise contrasts for the ASV data of those metrics.

**Fig. 4.**
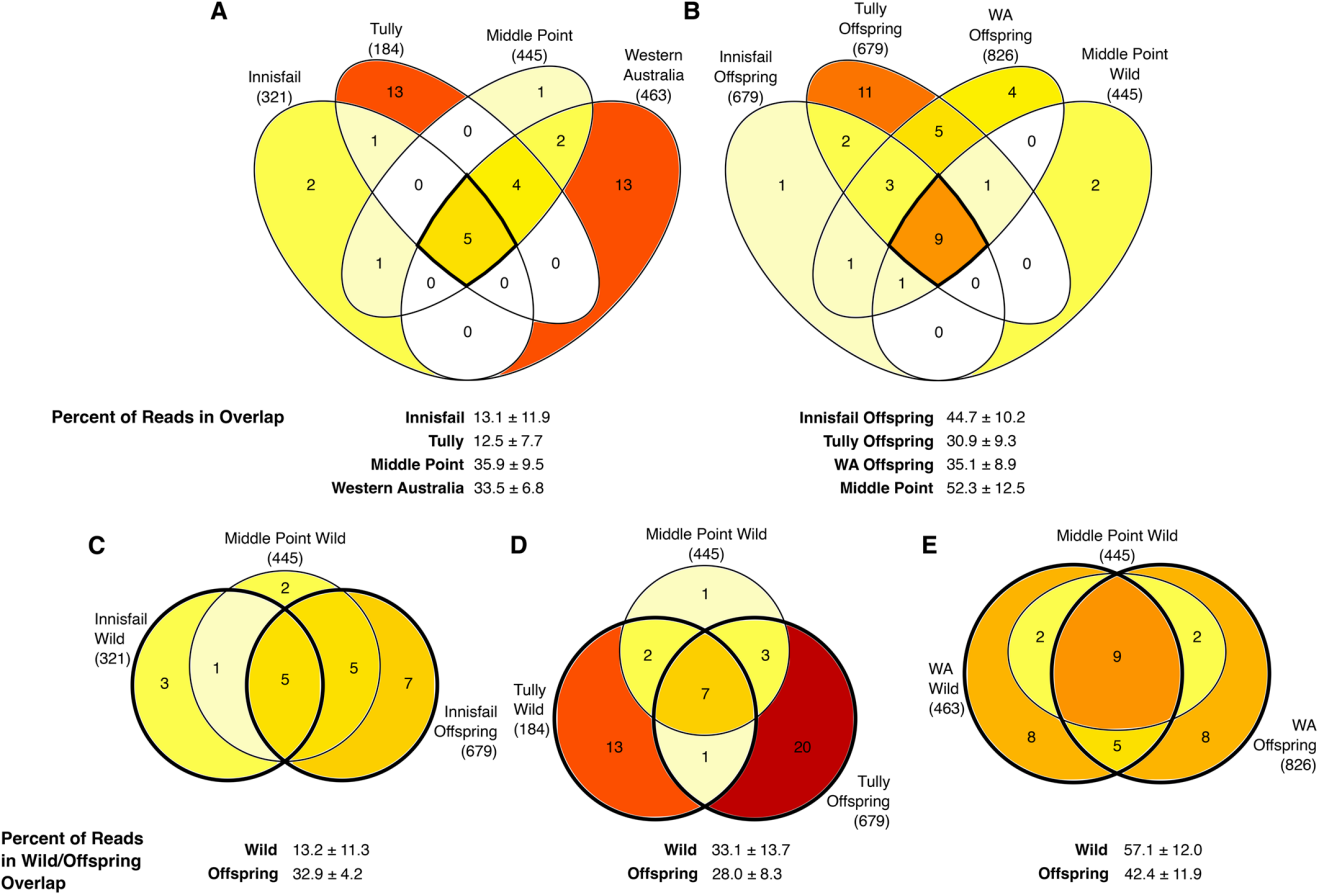
**Venn diagrams representing core skin bacterial communities (ASVs with 100% prevalence) on groups of cane toads (sample sizes per group are given in Table 1).** Comparisons are among (A) wild sites and (B) toads at Middle Point. Common-garden offspring are further compared with toads from their respective ancestral sites and wild toads from Middle Point for each of (C) Innisfail, (D) Tully, and (E) Western Australia. Numbers and shading in the diagrams indicate number of microbes in the segment. Values in parentheses are the total number of ASVs present in samples from that group of toads. Below each diagram are percentages of reads represented by core microbes shared between groups of interest (average±s.d.). These overlaps of interest are highlighted in the diagrams with bold outlines. Relocated parent toads were excluded from Venn diagrams due to their small sample sizes.

Supporting our previous results (Weitzman et al., 2019), we found little differentiation of bacterial communities on wild free-ranging toads by site based on alpha diversity metrics. The site×captivity interaction term was not significant for Shannon diversity or evenness, so we did not run pairwise contrasts among the toad groups for those diversity metrics (Table 2, Figs 2 and 3). There were, however, consistent differences among the four wild sampling sites in all four beta diversity metrics, with the only similarity being between the two Queensland sites (Innisfail and Tully) in weighted UniFrac. Many of these differences were influenced by differences in dispersion (Table 2, Fig. 3; Fig. S2). Five ASVs were found in every wild-toad sample, representing on average 27% of the reads per sample (Fig. 4A). Toads from Innisfail and Middle Point had relatively depauperate core communities compared to toads from Tully and Western Australia.

**Table 2.**
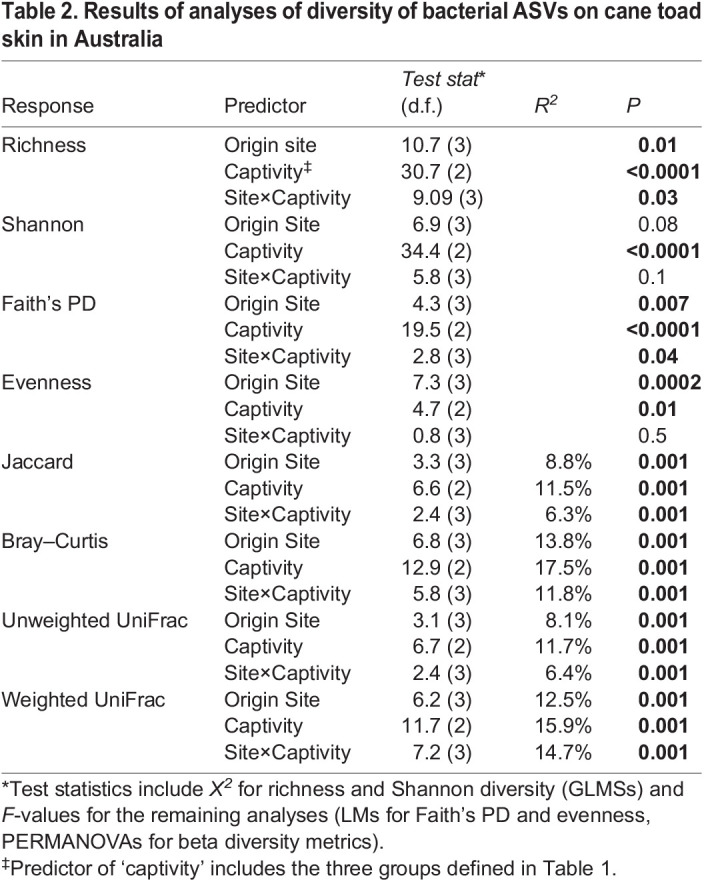
Results of analyses of diversity of bacterial ASVs on cane toad skin in Australia

### Toad relocation

Bacterial assemblages on toads relocated from Tully (Queensland) to Middle Point (Northern Territory) had similar alpha diversity metrics to their offspring, as well as, to wild toads from Middle Point and Tully, but they generally had different beta diversity (Table 2; Figs. 2, 3, Fig. S2). Samples from toads relocated from Innisfail (Queensland), on the other hand, had similar alpha diversity to the Innisfail common-garden offspring, but differed from wild toads at Middle Point and Innisfail in richness and phylogenetic diversity.

### Common-garden and toad captivity

Bacterial assemblages on common-garden offspring from parents taken from three collection sites did not differ significantly from each other in alpha diversity and rarely differed in beta diversity (Table 2; Figs. 2, 3). Groups of offspring all differed in Bray­–Curtis metrics, and those with Western Australia and Innisfail parentage differed in Jaccard metrics, though the two UniFrac metrics were not significant in any pairwise permutational multivariate analysis of variance (PERMANOVA). These assemblages generally did, however, differ from those seen on wild toads from nearby Middle Point, particularly in beta diversity (all metrics). Similarly, toad bacterial communities on wild individuals from the toads’ ancestral sites were similar to those on common-garden offspring in terms of alpha diversity but not beta diversity (all metrics).

Overlap in core bacteria (taxa with 100% prevalence and >0.1% average relative abundance among toads in a group) on common-garden toads compared to wild toads from ancestral sites and nearby Middle Point varied among the three site groups (Fig. 4B), though generally at least a quarter of the reads on common-garden toads were from ASVs shared with their wild ancestral counterparts. Common-garden Tully offspring and wild toads collected at Tully both had many core ASVs not seen in other groups’ core communities (Fig. 4). Many of the core ASVs on common-garden offspring were shared among offspring groups as well as with wild Middle Point toads (Fig. 4). The only captive-reared toads that exhibited a stronger core community overlap with wild toads from their ancestral location than with other common-garden offspring or wild toads at Middle Point were offspring from Western Australian adult toads.

These similarities in microbiomes on common-garden toads whose parents came from different collection sites suggest an effect of captivity or location on skin communities. Indeed, captivity status was a significant predictor of all of the diversity metrics (Table 2), accounting for 11.5­–17.5% of the variation in beta diversity.

### ASVs versus OTUs

We expected to find stronger origin site×captivity effects when we relaxed microbial identification from sequence variants to OTU clustering of similar bacterial reads. Instead, we saw few differences in results between analyses of ASVs and OTUs (Table S1, Fig. 3). With OTUs, significant differences emerged among free-ranging toad sites. Additional changes (flips in significance) arose between captive toads and relevant wild-caught toads, with two switches in significance toward similarity between groups of common-garden offspring toads.

Unsurprisingly, relaxing our criteria from core ASVs to core OTUs accounted for larger proportions of reads representing overlapping core taxa (Figs. S4–S6). Notably, the overlap in core bacteria between wild Tully toads and common-garden offspring from Tully on average accounted for 23.8% (±13.3% SD) more reads in core OTUs when compared with core ASVs.

## DISCUSSION

In this experiment, we sampled skin microbiomes of three groups of cane toads: (1) wild cane toads collected at sites across tropical Australia; (2) toads from those sites after being held in captivity for three years at a single site; and (3) the mature offspring of those captive adults, raised under standardised conditions. Despite differing microbial assemblages in wild-caught toads from different geographic locations, microbiomes of captive toads (both translocated adults and captive-reared offspring) were broadly similar to each other.

As predicted from our own preliminary results from these samples (Weitzman et al., 2019) and studies on other amphibians (Christian et al., 2018; Hughey et al., 2017; Kueneman et al., 2014), bacterial composition differed among wild cane toads from geographically separated sites. Nearly every measure of beta diversity differed between sample locations, although these results also include differences in dispersion between groups of communities. Despite these differences, approximately one-quarter of the reads in wild-toad samples represented ASVs present in every wild toad sampled.

Although there were significant differences in community structure among sample locations, it is apparent in principal coordinates analysis and relative abundances of taxa (Fig. 1; Figs S2, S3) that bacterial samples from Queensland toads (Innisfail and Tully) differed from those on toads collected at the other two sites. Conversely, we found strong similarity in bacteria on toads from Middle Point, Western Australia, and captive and common-garden offspring at Middle Point, particularly in Bray­–Curtis and weighted UniFrac space. This result may be due to proximity: the two Queensland sites were separated by ∼50 km, but additionally the Queensland sites were cooler and wetter at the time of sampling than the sites farther west. Interestingly, two bacterial genera common in cane toads and other frogs in the wet tropics, *Pseudomonas* and *Acinetobacter* (Abarca et al., 2018; Nava-González et al., 2021) were abundant in our two Queensland sites but not in the other groups of toads, suggesting that these taxa may thrive in wetter conditions. The lack of similar differences in captive-raised offspring in the common-garden experiment as those found among the wild toads suggest that community composition may be driven by local environments rather than by evolved host traits.

Contrary to our predictions, we found no indication of an influence of parental collection site on bacterial microbiomes on our common-garden offspring toads. Despite many morphological, physiological, and behavioural traits evolving among populations of toads in their invaded range of Australia (Gruber et al., 2017; Hudson et al., 2018; Rollins et al., 2015), the relatively short timeframe (<100 years and generations) since the species’ introduction to Australia has not resulted in detectable heritable differences in skin bacterial assemblies. In another frog host, Jani and Briggs (2018) found that bacterial microbiomes on Sierra Nevada yellow-legged frogs (*Rana sierrae*) collected as eggs and tadpoles from two nearby populations in California, USA, were still affected by source population after being raised in a co-housed environment. In the present study, however, toads bred in captivity and raised in semi-natural enclosures had similar microbial communities, different from those on wild-sampled individuals (including from parental populations). This result suggests that bacterial assemblages on a toads’ skin in tropical Australia are affected more by local environments, or by the host's phenotypic plastic responses to those environments, than by heritable differences in host physiology that drive microbial diversity. Perhaps over more generations or stronger selective pressures, evolution of the mucosal role in bacterial recruitment will produce detectable effects.

The mechanisms of how amphibian skin and its secretions select for bacterial communities are not well understood, and these mechanisms likely also vary among groups of amphibians. In some frogs, specific anti-microbial peptides (AMPs) secreted from skin glands correlate with cutaneous bacteria (Davis et al., 2017), and studies suggest that AMPs may have an important role in cutaneous bacterial assemblages (Küng et al., 2014; Loudon et al., 2020). Although true toads in the family Bufonidae are not known to produce AMPs *per se* (Conlon et al., 2009), secretions from toads’ skin glands contain diverse compounds, and those secreted by cane toads have antibacterial properties (de Medeiros et al., 2019). In cane toads and closely related congeners, variation in parotoid gland secretion composition has a phylogenetic signal (Maciel et al., 2006, 2010). The lack of a parental site-level signal in our common-garden toad skin microbiomes might suggest that either cane toad secretions do not strongly influence skin bacteria, or the composition of these secretions has not dramatically changed during the cane toad expansion across Australia.

As found in studies of other amphibians (Kueneman et al., 2022), captivity affected the cane toad skin bacteria. Only small sample sizes of captive parental toads were available at the time of sampling, limiting our statistical power and the extent to which we can interpret results from parental toads’ communities. Nevertheless, bacteria on toads relocated from Queensland generally clustered in principal coordinate space with others at Middle Point and away from communities on wild-caught Queensland toads (Figs. S2, S3), having bacteria more similar to the common-garden offspring and suggesting shifts due to captivity. These changes were likely affected by changes in microhabitat as well as in broader environmental and climatic factors. Bacterial sampling across time would give us more detailed information on the strength of captivity effects in this system. In a recent meta-analysis, Kueneman et al. (2022) found effects of captivity on skin communities of 18 frog and salamander species. Individuals housed in semi-natural conditions, such as those used in our toad experiment, often had distinct communities from wild individuals (Kueneman et al., 2022). A strong effect of local conditions should have resulted in similar microbiomes for captive toads housed at Middle Point and wild Middle Point toads, but this was not the case. This result suggests that either the offspring of toads from Queensland and Western Australia select for similar communities (which is not implied by wild toads from those sites), or our semi-natural enclosures did not provide the same microbes as those encountered by wild toads in the area. Interestingly, wild toads at Middle Point had fewer bacterial types on their skin than did the common-garden offspring.

Among the bacterial ASVs found on the wild toads, most (689 of the 759 ASVs on wild toads) were not present on individuals from all four sites sampled. Plausibly, the sites had different bacteria available to colonize the toads. Skin bacteria may be acquired from environmental taxa and vertical and horizontal transmission (Becker et al., 2014; McGrath-Blaser et al., 2021; Rebollar et al., 2016; Walke et al., 2011, 2014). As environmental bacteria differ across space and substrate characteristics (Belotte et al., 2003; Fierer et al., 2007; García-García et al., 2019), affecting the bacteria available to colonise the toads, we predicted that loosening the clustering requirements to OTUs (97% similarity) from ASVs would reveal stronger patterns. Overlap of core ASVs accounted for over a quarter of the reads on wild toads, and analysing OTUs did not dramatically change these values. By grouping similar ASVs into OTUs, the proportion of reads from core taxa increased by just ∼1%. It should further be noted that our cut-off for inclusion in core communities was chosen because of our uneven sampling effort and small sample sizes, which may have excluded some prevalent ASVs with low relative abundance.

Our study identifies additional questions regarding the role of microdiversity in these bacterial communities. A larger sampling of toads could clarify patterns; for example, OTUs with high microdiversity may persist over a greater range of environments (García-García et al., 2019). In particular, it would be valuable to expand sampling to other sites where the toad has been introduced to investigate persistence of bacterial types among even greater distances and introduction histories. For example, one of the most common and abundant bacterial genera in our samples, *Niabella*, also is abundant on cane toads in Puerto Rico (Abarca et al., 2018). Although describing bacterial communities using relatively short reads of a conserved region of the bacterial genome (i.e. 16S rRNA gene) has become commonplace and thus relatively inexpensive and computationally streamlined, other methods may be better suited for identifying microdiversity within and among communities. However, as noted by Okazaki et al. (2021), who used long-read amplicon sequencing to tackle questions regarding freshwater bacterioplankton microdiversity, this and other methods present challenges of higher costs of sequencing for lower read depth.

Our sampling was sufficient to show that skin microbiomes on invasive cane toads are affected by local environmental conditions, but we need more extensive sampling to tease apart other influences. The bacterial assemblages that colonise toad skin likely are affected by a complex interplay between local environments and host traits, providing an excellent model system with which to explore the role of multiple interacting factors in driving microbial diversity.

## MATERIALS AND METHODS

Toads from two sites in Queensland [Innisfail (17.53 °S, 146.03 °E), Tully (17.93 °S, 145.92 °E)] and four sites near the invasion front in Western Australia [El Questro (16.01 °S, 127.98 °E), Oombulgurri (15.18 °S, 127.85 °E), Wyndham (15.47 °S, 128.10 °E), Purnululu (17.53 °S, 128.41 °E)] were transported to Middle Point, Northern Territory (12.58 °S, 131.31 °E) in November­–December 2013. Toad spawning was induced by injecting pairs of parents from each site with artificial gonadotropins (Brannelly et al., 2019). Tadpoles were reared in outdoor tanks until metamorphosis. After metamorphosis, common-garden offspring were individually toe-clipped for identification and housed outdoors in 700 L containers equipped with automated sprinklers to provide water and overhead lights to attract nocturnal insects for food. Parental toads were also housed under these conditions, but in separate containers from offspring. As they grew, offspring were regularly measured and size-assorted into housing groups (to prevent cannibalism), random to ancestral origin. Thus, offspring were exposed to several different, sometimes temporary, enclosure-mates over the subsequent years. Further details on the common-garden breeding and rearing can be found in (Hudson et al., 2016).

When the offspring were approximately 3 years post-metamorphosis (September 2017), we collected skin swab samples from common-garden offspring and the remaining captive parents at Middle Point, along with wild toads from four sites across Australia (Table 1; Innisfail and Tully in Queensland, Middle Point in the Northern Territory, and near the invasion front in Western Australia [Marlgu Billabong near Wyndham (15.55 °S, 128.26 °E)]. For a map of wild-caught sites, see Appendix Fig. 1 in Weitzman et al. (2019). Toads were rinsed with 100 ml high-purity water before swabbing with a sterile synthetic swab (Medical Wire and Equipment Company, Corsham, UK) as previously described (Christian et al., 2018; Weitzman et al., 2019). Swab samples were placed on ice in the field and frozen at −20 °C until DNA extraction using Norgen's Microbiome DNA Isolation Kit using the protocol for preserved samples, which is designed to extract DNA from both Gram-positive and Gram-negative cells (Norgen Biotek Corp., Thorold, ON, Canada). We sent 200 ng dried DNA per sample to ACE Sequencing Service at the Australian Centre for Ecogenomics (University of Queensland) for Illumina MiSeq sequencing (2×300 bp chemistry) targeting the V4 variable region of bacterial 16S rRNA using the 515F/806R primers (Caporaso et al., 2012).

Using the dada2 package in R v4.0.2 in Rstudio v1.3.1093 (Callahan et al., 2016; R Development Core Team, 2015; RStudio Team, 2020), demultiplexed paired-end reads were quality-filtered, reads were trimmed (260 bp forward, 180 bp reverse), primers were removed, and maximum expected errors allowed were 2 or 5 for forward and reverse reads, respectively. We retained merged reads between 250–257 bp, then removed chimeras and assigned taxonomy to the remaining amplicon sequence variants (ASVs) based on the Silva v138.1 database (Quast et al., 2012; Yilmaz et al., 2014). From the resultant data, we retained bacterial ASVs using QIIME2 (Bolyen et al., 2019), further removing reads assigned as mitochondria or chloroplast. For OTU comparisons, we then clustered bacterial ASVs into OTUs with 97% similarity using vsearch (Rognes et al., 2016). While two DNA extraction controls were included in the sequencing run, they had <2000 reads after processing and were removed from the analysed dataset. One additional sample that failed in sequencing with very low reads was removed from analyses (Table 1).

After evaluation of rarefaction curves in QIIME2, we measured richness and alpha diversity metrics (Faith's phylogenetic diversity, Shannon's diversity, and Pielou's evenness) from reads rarefied to 14,459 reads per sample, removing six samples from alpha diversity analyses (Table 1). Beta diversity metrics, including weighted and unweighted UniFrac distances, Jaccard index, and Bray–Curtis distances, were calculated based on the entire unrarefied dataset, with Bray–Curtis distances calculated from a feature table of proportional relative abundances (as is suggested as the best practice by McKnight et al., 2019) in the vegan package in R (Oksanen et al., 2020). The other three metrics are either based on presence/absence data (unweighted UniFrac, Jaccard) or inherently work with proportional data (weighted UniFrac). UniFrac distances incorporate phylogenetic data of the bacteria, for which we used a mid-point rooted tree computed in QIIME2.

Lastly, we identified core bacteria to assess overlaps in prevalent taxa between groups of toads, using a conservative estimate of core communities to avoid overestimating the importance of transient taxa. We defined core bacteria as those that were present in 100% of the samples within each group of toads, including only those that accounted for, on average, at least 0.1% of the relative abundance of the bacteria on those toads. The relative abundance cut-off was included to remove rare, transient taxa and to counteract effects of different sampling depths without removing data with rarefaction (Neu et al., 2021). We used Venn diagrams to visualise the overlap among toad groups of core ASVs and OTUs.

### Statistics

For analyses, toads were grouped by captivity status (wild, captive parents, common-garden offspring) and toad origin/ancestral site (four sites) as in Table 1. At the time of sampling the common-garden toads, there were too few offspring remaining from any one Western Australia site parentage to focus the study on offspring from one site, requiring pooling the offspring from invasion front toads into a single common-garden group. We make direct comparisons between this Western Australia offspring pool and wild toads sampled from a site near one of the parentage sites to assess invasion front bacterial dynamics.

R v4.0.2 in Rstudio v1.3.1093 (R Core Team, 2020; RStudio Team, 2020) was used for analyses. To address differences among microbial communities on toads from multiple wild and captive sources, all analyses, unless otherwise stated, were run with toad origin/ancestral site, captivity status, and their interaction as predictor variables. We then used *post-hoc* analyses to identify pairwise differences among the comparisons of interest.

We used generalised linear models to analyse richness [negative binomial in the MASS package (Venables and Ripley, 2002)] and Shannon diversity (Gamma with log-link) and linear models to analyse Pielou's evenness and Faith's phylogenetic diversity. We calculated pairwise contrasts in the emmeans package, which adjusts *P*-values using the Tukey method (Lenth et al., 2022). Beta diversity metrics were analysed with PERMANOVAs (999 permutations) and analysis of beta dispersion in the vegan package, with pairwise PERMANOVAs (999 permutations) conducted with the RVAideMemoire package and *P*-values adjusted using the Benjamini–Hochberg method (Hervé, 2022). To address all possible pairwise tests, we assessed *post-hoc* contrasts with a group predictor variable that split the samples into the nine groups outlined in Table 1. We visualised beta diversity with principal coordinates analysis calculated in the ape package (Paradis and Schliep, 2019).

We used Wilcoxon signed rank tests to compare the relative abundances comprised of overlapping core ASVs versus core OTUs to discern patterns in groups of wild toads, common-garden offspring, and common-garden and ancestral toads separately.

## Supplementary Material

10.1242/biolopen.059641_sup1 Supplementary information

## References

[BIO059641C1] Abarca, J. G., Zuniga, I., Ortiz-Morales, G., Lugo, A., Viquez-Cervilla, M., Rodriguez-Hernandez, N., Vázquez-Sánchez, F., Murillo-Cruz, C., Torres-Rivera, E. A., Pinto-Tomás, A. A. et al. (2018). Characterization of the skin microbiota of the cane toad *Rhinella cf. marina* in Puerto Rico and Costa Rica. *Front. Microbiol.* 8, 2624. 10.3389/fmicb.2017.02624 29354109 PMC5760547

[BIO059641C2] Adair, K. L. and Douglas, A. E. (2017). Making a microbiome: the many determinants of host-associated microbial community composition. *Curr. Opin. Microbiol.* 35, 23-29. 10.1016/j.mib.2016.11.002 27907842

[BIO059641C3] Alford, R. A., Brown, G. P., Schwarzkopf, L., Phillips, B. L. and Shine, R. (2009). Comparisons through time and space suggest rapid evolution of dispersal behaviour in an invasive species. *Wild Res.* 36, 23-28. 10.1071/WR08021

[BIO059641C4] Becker, M. H., Richards-Zawacki, C. L., Gratwicke, B. and Belden, L. K. (2014). The effect of captivity on the cutaneous bacterial community of the critically endangered Panamanian golden frog (*Atelopus zeteki*). *Biol. Conserv.* 176, 199-206. 10.1016/j.biocon.2014.05.029

[BIO059641C5] Belasen, A. M., Riolo, M. A., Bletz, M. C., Lyra, M. L., Toledo, L. F. and James, T. Y. (2021). Geography, host genetics, and cross-domain microbial networks structure the skin microbiota of fragmented Brazilian Atlantic forest frog populations. *Ecol. Evol.* 11, 9293-9307. 10.1002/ece3.7594 34306622 PMC8293785

[BIO059641C6] Belotte, D., Curien, J. B., Maclean, R. C. and Bell, G. (2003). An experimental test of local adaptation in soil bacteria. *Evolution* 57, 27-36. 10.1111/j.0014-3820.2003.tb00213.x 12643565

[BIO059641C7] Bolyen, E., Rideout, J. R., Dillon, M. R., Bokulich, N. A., Abnet, C. C., Al-Ghalith, G. A., Alexander, H., Alm, E. J., Arumugam, M., Asnicar, F. et al. (2019). Reproducible, interactive, scalable and extensible microbiome data science using QIIME 2. *Nat. Biotechnol.* 37, 852-857. 10.1038/s41587-019-0209-9 31341288 PMC7015180

[BIO059641C8] Brannelly, L. A., Ohmer, M. E. and Richards-Zawacki, C. L. (2019). Artificial reproduction using leuprolide acetate in the frog *Rana pipiens*. *Herpetol. J.* 29, 125-130. 10.33256/hj29.2.125130

[BIO059641C9] Brown, G. P., Phillips, B. L., Dubey, S. and Shine, R. (2015). Invader immunology: invasion history alters immune system function in cane toads (*Rhinella marina*) in tropical Australia. *Ecol. Lett.* 18, 57-65. 10.1111/ele.12390 25399668

[BIO059641C10] Callahan, B. J., Mcmurdie, P. J., Rosen, M. J., Han, A. W., Johnson, A. J. A. and Holmes, S. P. (2016). DADA2: high-resolution sample inference from Illumina amplicon data. *Nat. Methods* 13, 581-583. 10.1038/nmeth.3869 27214047 PMC4927377

[BIO059641C11] Caporaso, J. G., Lauber, C. L., Walters, W. A., Berg-Lyons, D., Huntley, J., Fierer, N., Owens, S. M., Betley, J., Fraser, L., Bauer, M. et al. (2012). Ultra-high-throughput microbial community analysis on the Illumina HiSeq and MiSeq platforms. *ISME J.* 6, 1621-1624. 10.1038/ismej.2012.8 22402401 PMC3400413

[BIO059641C12] Christian, K., Weitzman, C., Rose, A., Kaestli, M. and Gibb, K. (2018). Ecological patterns in the skin microbiota of frogs from tropical Australia. *Ecol. Evol.* 8, 10510-10519. 10.1002/ece3.4518 30464823 PMC6238143

[BIO059641C13] Conlon, J. M., Iwamuro, S. and King, J. D. (2009). Dermal cytolytic peptides and the system of innate immunity in anurans. *Ann. NY Acad Sci.* 1163, 75-82. 10.1111/j.1749-6632.2008.03618.x 19456329

[BIO059641C14] Davis, L. R., Bigler, L. and Woodhams, D. C. (2017). Developmental trajectories of amphibian microbiota: response to bacterial therapy depends on initial community structure. *Environ. Microbiol.* 19, 1502-1517. 10.1111/1462-2920.13707 28229543

[BIO059641C15] De Medeiros, D. S., Rego, T. B., Dos Santos, A. P. A., Pontes, A. S., Moreira-Dill, L. S., Matos, N. B., Zuliani, J. P., Stábeli, R. G., Teles, C. B., Soares, A. M. et al. (2019). Biochemical and biological profile of parotoid secretion of the Amazonian *Rhinella marina* (Anura: Bufonidae). *Biomed. Res. Int.* 2019, 2492315. 10.1155/2019/4209743 31214612 PMC6535847

[BIO059641C16] Ellison, S., Rovito, S., Parra-Olea, G., Vásquez-Almazán, C., Flechas, S. V., Bi, K. and Vredenburg, V. T. (2019). The influence of habitat and phylogeny on the skin microbiome of amphibians in Guatemala and Mexico. *Microb. Ecol.* 78, 257-267. 10.1007/s00248-018-1288-8 30467714

[BIO059641C17] Fierer, N., Bradford, M. A. and Jackson, R. B. (2007). Toward an ecological classification of soil bacteria. *Ecology* 88, 1354-1364. 10.1890/05-1839 17601128

[BIO059641C18] García-García, N., Tamames, J., Linz, A. M., Pedrós-Alió, C. and Puente-Sánchez, F. (2019). Microdiversity ensures the maintenance of functional microbial communities under changing environmental conditions. *ISME J.* 13, 2969-2983. 10.1038/s41396-019-0487-8 31417155 PMC6864100

[BIO059641C19] Gruber, J., Brown, G., Whiting, M. J. and Shine, R. (2017). Geographic divergence in dispersal-related behaviour in cane toads from range-front versus range-core populations in Australia. *Behav. Ecol. Sociobiol.* 71, 38. 10.1007/s00265-017-2266-8

[BIO059641C20] Hervé, M. (2022). RVAideMemoire: Testing and Plotting Procedures for Biostatistics. *R package version 0.9-81-2*. Available at: https://CRAN.R-project.org/package=RVAideMemoire

[BIO059641C21] Hudson, C. M., Brown, G. P. and Shine, R. (2016). It is lonely at the front: contrasting evolutionary trajectories in male and female invaders. *R. Soc. Open Sci.* 3, 160687. 10.1098/rsos.160687 28083108 PMC5210690

[BIO059641C22] Hudson, C. M., Brown, G. P., Stuart, K. and Shine, R. (2018). Sexual and geographical divergence in head widths of invasive cane toads, *Rhinella marina* (Anura: Bufonidae), is driven by both rapid evolution and plasticity. *Biol. J. Linn. Soc.* 124, 188-199. 10.1093/biolinnean/bly040

[BIO059641C23] Hughey, M. C., Pena, J. A., Reyes, R., Medina, D., Belden, L. K. and Burrowes, P. A. (2017). Skin bacterial microbiome of a generalist Puerto Rican frog varies along elevation and land use gradients. *PeerJ* 5, e3688. 10.7717/peerj.3688 28875068 PMC5580383

[BIO059641C24] Jani, A. J. and Briggs, C. J. (2018). Host and aquatic environment shape the amphibian skin microbiome but effects on downstream resistance to the pathogen *Batrachochytrium dendrobatidis* are variable. *Front. Microbiol.* 9, 487. 10.3389/fmicb.2018.00487 29619014 PMC5871691

[BIO059641C25] Kohl, K. D. (2020). Ecological and evolutionary mechanisms underlying patterns of phylosymbiosis in host-associated microbial communities. *Philos. Trans. R. Soc. Lond. B* 375, 20190251. 10.1098/rstb.2019.0251 32200746 PMC7133527

[BIO059641C26] Kosmala, G. K., Brown, G. P. and Shine, R. (2020). Thin-skinned invaders: geographic variation in the structure of the skin among populations of cane toads (*Rhinella marina*). *Biol. J. Linn. Soc.* 131, 611-621. 10.1093/biolinnean/blaa128

[BIO059641C27] Kueneman, J. G., Parfrey, L. W., Woodhams, D. C., Archer, H. M., Knight, R. and Mckenzie, V. J. (2014). The amphibian skin-associated microbiome across species, space and life history stages. *Mol. Ecol.* 23, 1238-1250. 10.1111/mec.12510 24171949

[BIO059641C28] Kueneman, J., Bletz, M., Becker, M., Gratwicke, B., Garcés, O. A., Hertz, A., Holden, W. M., Ibáñez, R., Loudon, A. and Mckenzie, V. et al. (2022). Effects of captivity and rewilding on amphibian skin microbiomes. *Biol. Conserv.* 271, 109576. 10.1016/j.biocon.2022.109576

[BIO059641C29] Küng, D., Bigler, L., Davis, L. R., Gratwicke, B., Griffith, E. and Woodhams, D. C. (2014). Stability of microbiota facilitated by host immune regulation: informing probiotic strategies to manage amphibian disease. *PLoS One* 9, e87101. 10.1371/journal.pone.0087101 24489847 PMC3906108

[BIO059641C30] Lenth, R (2022). emmeans: Estimated Marginal Means, aka Least-Squares Means. *R package version 1.8.3*. Available at: https://CRAN.R-project.org/package=emmeans

[BIO059641C31] Loudon, A. H., Kurtz, A., Esposito, E., Umile, T. P., Minbiole, K. P. C., Parfrey, L. W. and Sheafor, B. A. (2020). Columbia spotted frogs (*Rana luteiventris*) have characteristic skin microbiota that may be shaped by cutaneous skin peptides and the environment. *FEMS Microbiol. Ecol.* 96, fiaa168. 10.1093/femsec/fiaa168 32815986

[BIO059641C32] Maciel, N. M., Schwartz, C. A., Colli, G. R., Castro, M. S., Fontes, W. and Schwartz, E. N. F. (2006). A phylogenetic analysis of species in the *Bufo crucifer* group (Anura: Bufonidae), based on indolealkylamines and proteins from skin secretions. *Biochem. Syst. Ecol.* 34, 457-466. 10.1016/j.bse.2006.01.005

[BIO059641C33] Maciel, N. M., Collevatti, R. G., Colli, G. R. and Schwartz, E. F. (2010). Late Miocene diversification and phylogenetic relationships of the huge toads in the *Rhinella marina* (Linnaeus, 1758) species group (Anura: Bufonidae). *Mol. Phylogenet. Evol.* 57, 787-797. 10.1016/j.ympev.2010.08.025 20813190

[BIO059641C34] Mcgrath-Blaser, S., Steffen, M., Grafe, T. U., Torres-Sánchez, M., Mcleod, D. S. and Muletz-Wolz, C. R. (2021). Early life skin microbial trajectory as a function of vertical and environmental transmission in Bornean foam-nesting frogs. *Animal Microbiome* 3, 83. 10.1186/s42523-021-00147-8 34930504 PMC8686334

[BIO059641C35] Mckenzie, V. J., Bowers, R. M., Fierer, N., Knight, R. and Lauber, C. L. (2012). Co-habiting amphibian species harbor unique skin bacterial communities in wild populations. *ISME J.* 6, 588-596. 10.1038/ismej.2011.129 21955991 PMC3280140

[BIO059641C36] Mcknight, D. T., Huerlimann, R., Bower, D. S., Schwarzkopf, L., Alford, R. A. and Zenger, K. R. (2019). Methods for normalizing microbiome data: an ecological perspective. *Methods Ecol. Evol.* 10, 389-400. 10.1111/2041-210X.13115

[BIO059641C37] Nava-González, B., Suazo-Ortuño, I., López, P. B., Maldonado-López, Y., Lopez-Toledo, L., Raggi, L., Parra-Olea, G., Alvarado-Díaz, J. and Gómez-Gil, B. (2021). Inhibition of *Batrachochytrium dendrobatidis* infection by skin bacterial communities in wild amphibian populations. *Microb. Ecol.* 82, 666-676. 10.1007/s00248-021-01706-x 33598748

[BIO059641C38] Neu, A. T., Allen, E. E. and Roy, K. (2021). Defining and quantifying the core microbiome: challenges and prospects. *Proc. Natl. Acad. Sci. USA* 118, e2104429118. 10.1073/pnas.2104429118 34862327 PMC8713806

[BIO059641C39] Okazaki, Y., Fujinaga, S., Salcher, M. M., Callieri, C., Tanaka, A., Kohzu, A., Oyagi, H., Tamaki, H. and Nakano, S. (2021). Microdiversity and phylogeographic diversification of bacterioplankton in pelagic freshwater systems revealed through long-read amplicon sequencing. *Microbiome* 9, 24. 10.1186/s40168-020-00974-y 33482922 PMC7825169

[BIO059641C40] Oksanen, J., Blanchet, F. G., Friendly, M., Kindt, R., Legendre, P., Mcglinn, D., Minchin, P. R., O'hara, R. B., Simpson, G. L., Solymos, P. et al. (2020). vegan: Community Ecology Package. *R package version 2.5–7*. Available at: https://CRAN.R-project.org/package=vegan

[BIO059641C41] Paradis, E. and Schliep, K. (2019). ape 5.0: an environment for modern phylogenetics and evolutionary analysis in R. *Bioinformatics* 35, 526-528. 10.1093/bioinformatics/bty633 30016406

[BIO059641C42] Phillips, B. L. (2009). The evolution of growth rates on an expanding range edge. *Biol. Lett.* 5, 802-804. 10.1098/rsbl.2009.0367 19605384 PMC2827979

[BIO059641C43] Quast, C., Pruesse, E., Yilmaz, P., Gerken, J., Schweer, T., Yarza, P., Peplies, J. and Glöckner, F. O. (2012). The SILVA ribosomal RNA gene database project: improved data processing and web-based tools. *Nucleic Acids Res.* 41, D590-D596. 10.1093/nar/gks1219 23193283 PMC3531112

[BIO059641C44] R Core Team (2020). *R: A Language and Environment for Statistical Computing (R Version 4.0. 2)*. R Foundation for Statistical Computing, Vienna, Austria. https://www.R-project.org/

[BIO059641C45] Rebollar, E. A., Simonetti, S. J., Shoemaker, W. R. and Harris, R. N. (2016). Direct and indirect horizontal transmission of the antifungal probiotic bacterium *Janthinobacterium lividum* on green frog (*Lithobates clamitans*) tadpoles. *Appl. Environ. Microbiol.* 82, 2457-2466. 10.1128/AEM.04147-15 26873311 PMC4959476

[BIO059641C46] Rognes, T., Flouri, T., Nichols, B., Quince, C. and Mahé, F. (2016). VSEARCH: a versatile open source tool for metagenomics. *PeerJ* 4, e2584. 10.7717/peerj.2584 27781170 PMC5075697

[BIO059641C47] Rollins, L. A., Richardson, M. F. and Shine, R. (2015). A genetic perspective on rapid evolution in cane toads (Rhinella marina). *Mol. Ecol.* 24, 2264-2276. 10.1111/mec.13184 25894012

[BIO059641C48] RStudio Team. (2020). *RStudio: Integrated Development Environment for R.* RStudio, PBC., Boston, MA, USA. http://www.rstudio.com/

[BIO059641C49] Selechnik, D., Richardson, M. F., Shine, R., Devore, J. L., Ducatez, S. and Rollins, L. A. (2019). Increased adaptive variation despite reduced overall genetic diversity in a rapidly adapting invader. *Front. Genet.* 10, 1221. 10.3389/fgene.2019.01221 31850072 PMC6901984

[BIO059641C50] Venables, W. N. and Ripley, B. D. (2002). *Modern Applied Statistics with S*, 4th edn. New York: Springer.

[BIO059641C51] Walke, J. B., Harris, R. N., Reinert, L. K., Rollins-Smith, L. A. and Woodhams, D. C. (2011). Social immunity in amphibians: evidence for vertical transmission of innate defenses. *Biotropica* 43, 396-400. 10.1111/j.1744-7429.2011.00787.x

[BIO059641C52] Walke, J. B., Becker, M. H., Loftus, S. C., House, L. L., Cormier, G., Jensen, R. V. and Belden, L. K. (2014). Amphibian skin may select for rare environmental microbes. *ISME J.* 8, 2207-2217. 10.1038/ismej.2014.77 24858782 PMC4992085

[BIO059641C53] Weitzman, C. L., Kaestli, M., Gibb, K., Brown, G. P., Shine, R. and Christian, K. (2019). Disease exposure and antifungal bacteria on skin of invasive cane toads, Australia. *Emerging Infect. Dis.* 25, 1770-1771. 10.3201/eid2509.190386 PMC6711215 31441753

[BIO059641C54] Yilmaz, P., Parfrey, L. W., Yarza, P., Gerken, J., Pruesse, E., Quast, C., Schweer, T., Peplies, J., Ludwig, W. and Glöckner, F. O. (2014). The SILVA and “all-species living tree project (LTP)” taxonomic frameworks. *Nucleic Acids Res.* 42, D643-D648. 10.1093/nar/gkt1209 24293649 PMC3965112

