# Supplementary material for: Geographic variation in bacterial assemblages on cane toad skin is influenced more by local environments than by evolved changes in host traits
Source: Biol Open. 2023 Feb 6;12(2):bio059641. doi: 10.1242/bio.059641 (PMC9932784; doi:10.1242/bio.059641)
Supplement: Supplementary information [file biolopen-12-059641-s1.pdf]

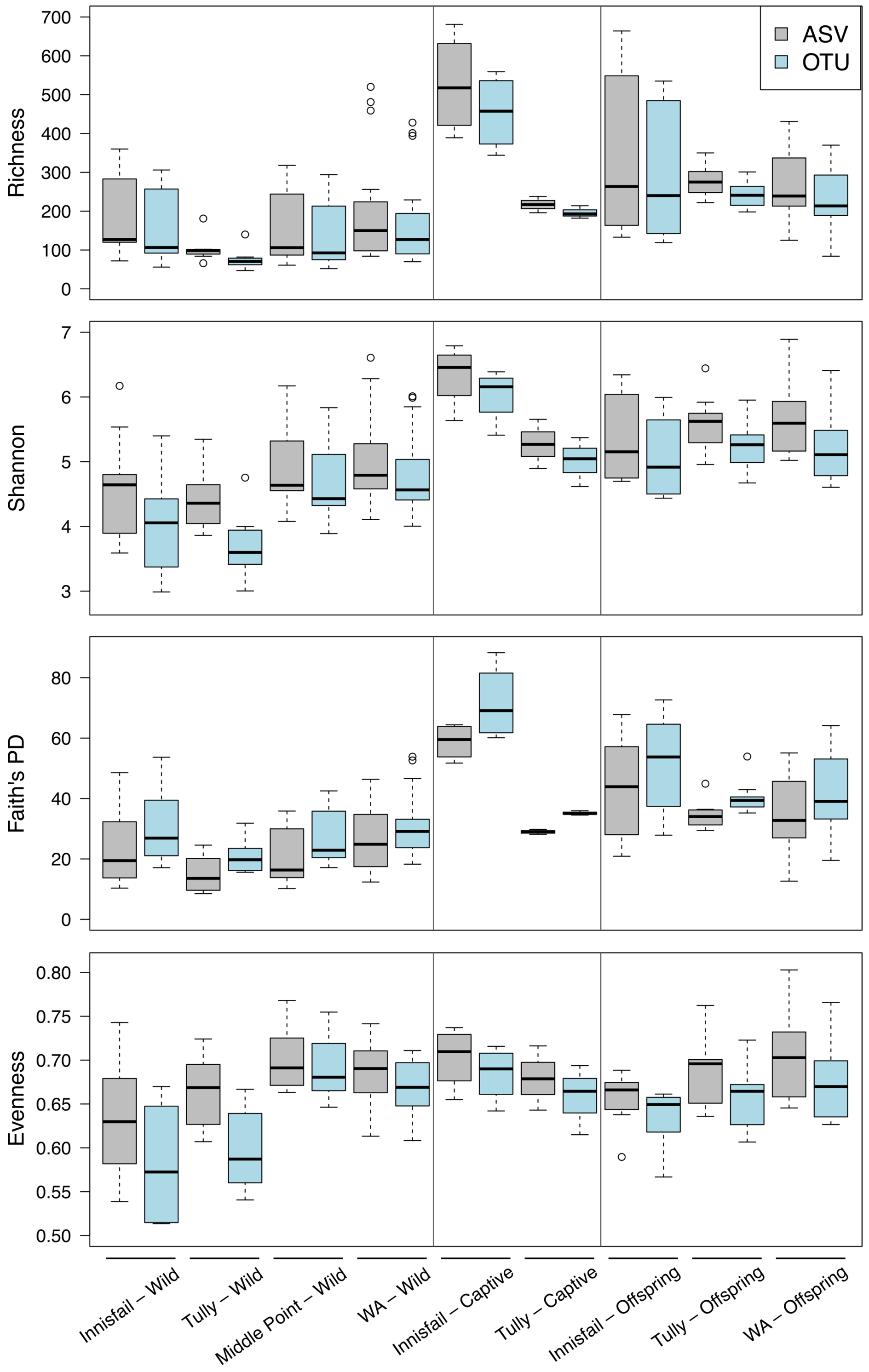

Fig. S1. Alpha diversity values in skin bacterial communities of captive and wild cane toads across Northern Australia. Boxplots show the median, interquartile range, reasonable range of the data, and outliers (open circles). Vertical lines separate data between wild toads, captive relocated toads, and common garden toad offspring. Grey = diversity of ASVs. Blue = diversity of OTUs.

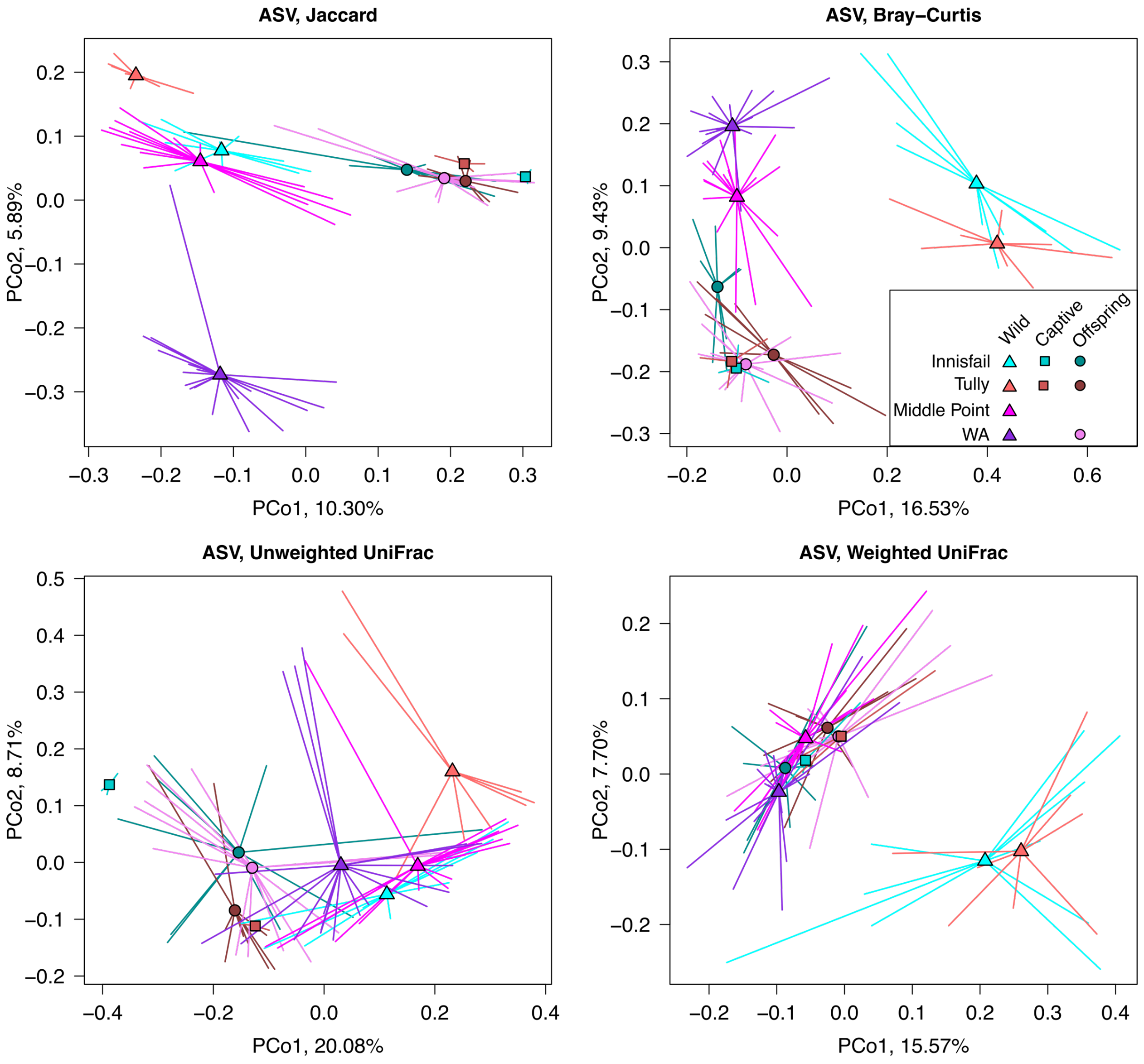

Fig. S2. Principal coordinates analyses of four beta diversity metrics of cane toad skin microbial ASVs from northern Australia. Points indicate averages within each group, with rays extending to individual sample points. Triangles = wild toads. Squares = toads brought into captivity. Circles = common garden offspring toads.

Biology Open • Supplementary information

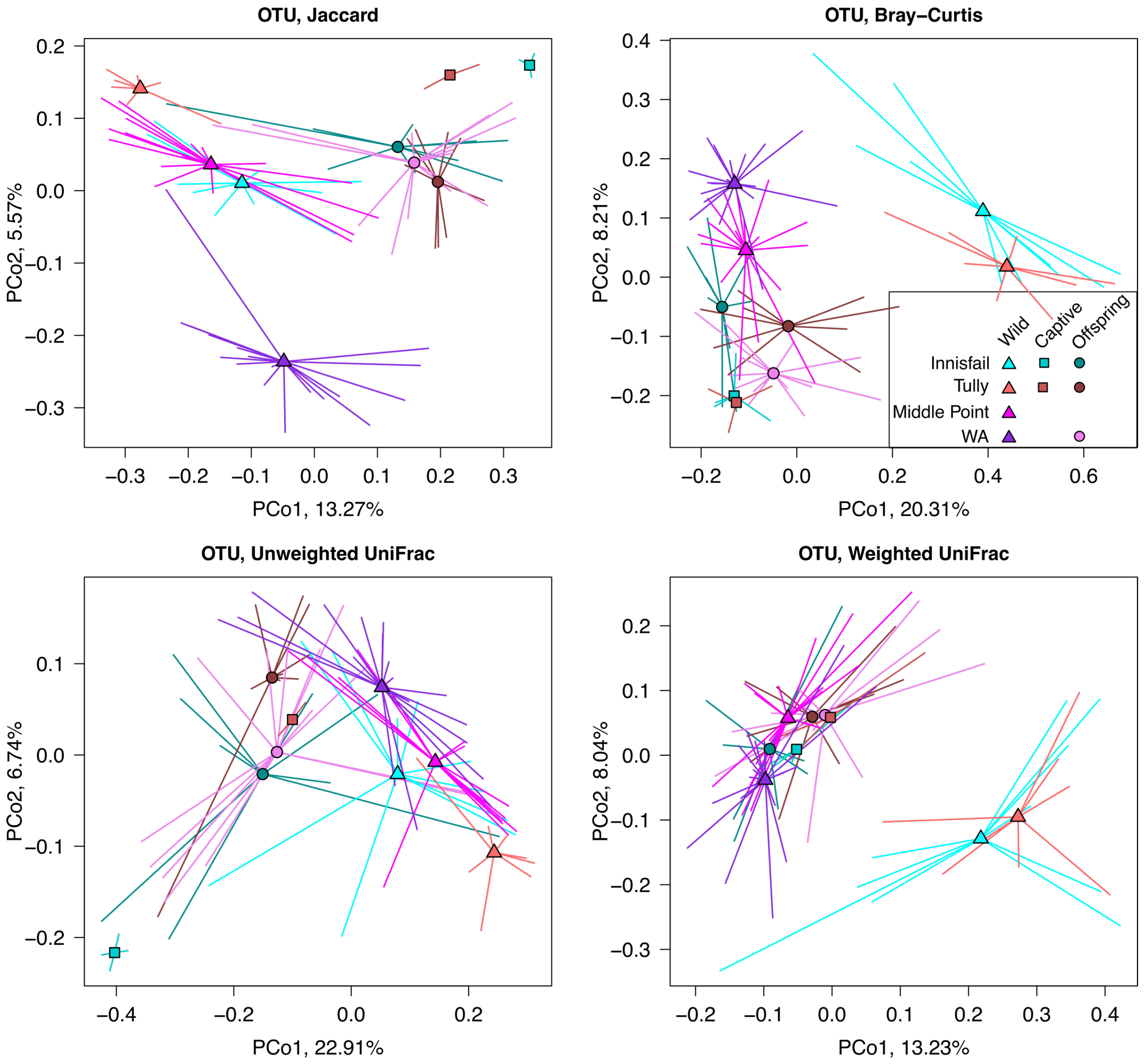

Fig. S3. Principal coordinates analyses of four beta diversity metrics of cane toad skin microbial OTUs from northern Australia. Points indicate averages within each group, with rays extending to individual sample points. Triangles = wild toads. Squares = toads brought into captivity. Circles = common garden offspring toads.

Biology Open • Supplementary information

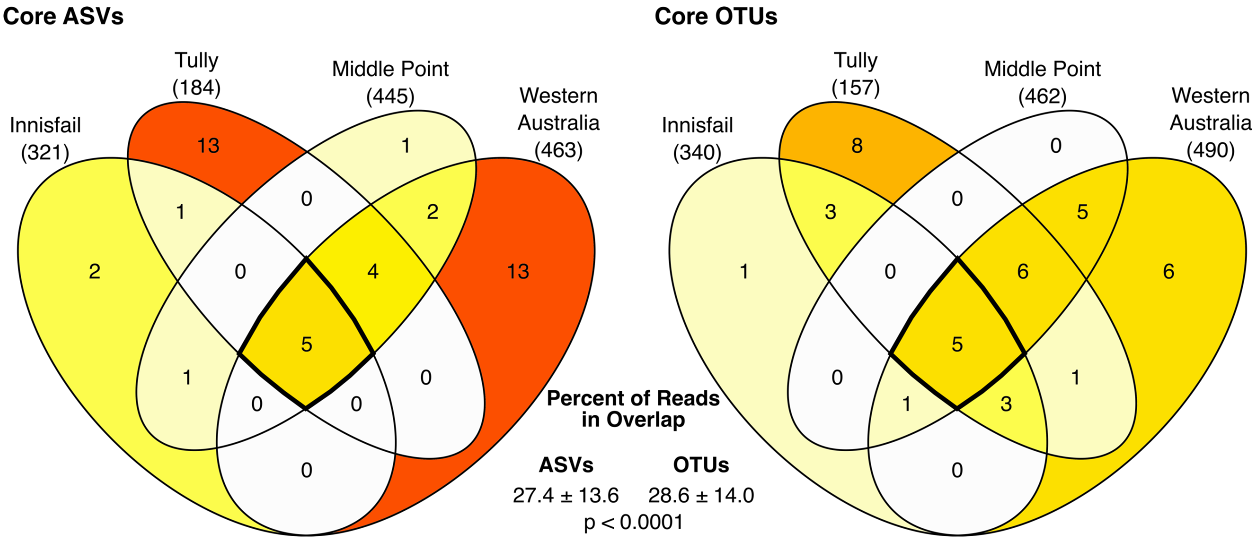

Fig. S4. Overlap of ASVs and OTUs in the core communities of wild toads from four sites in northern Australia. Numbers and shading in the diagrams indicate number of microbes in the segment. P-value from Wilcoxon signed rank test comparing proportion of reads in the overlap in ASVs versus OTUs.

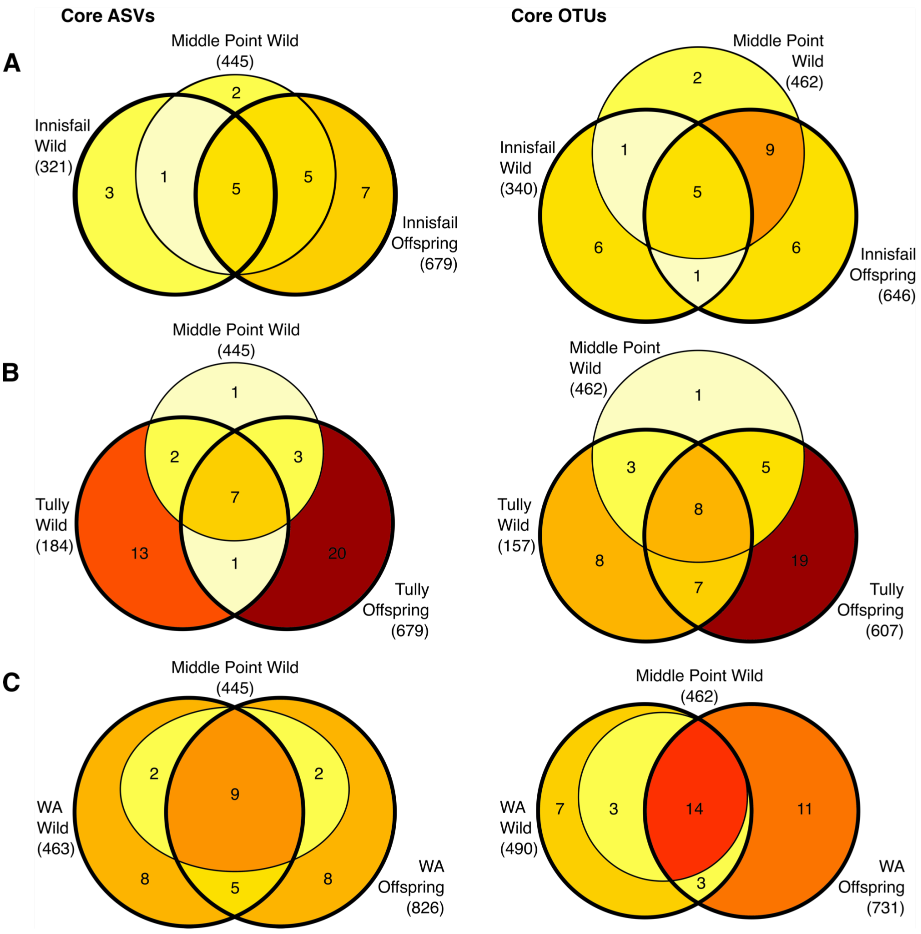

Percent of Reads in Wild/Offspring Overlap

(A)

	ASVs	OTUs
**Wild**	13.2 ± 11.3	17.0 ± 14.2
**Offspring**	32.9 ± 4.2	36.6 ± 3.5

p < 0.0001

(B)

	ASVs	OTUs
**Wild**	33.1 ± 13.7	58.4 ± 12.4
**Offspring**	28.0 ± 8.3	50.4 ± 11.2

p < 0.0001

(C)

	ASVs	OTUs
**Wild**	57.1 ± 12.0	66.7 ± 11.8
**Offspring**	42.4 ± 11.9	57.1 ± 12.3

p < 0.0001

Fig. S5. Venn diagrams representing overlap of ASVs and OTUs in the core communities (100% prevalence per group) of common garden toad offspring, toads at the respective ancestral sites, and nearby wild toads at Middle Point. (A) Innisfail, (B) Tully, and (C) Western Australia. Numbers and shading in the diagrams indicate number of microbes in the segment. Wild and common garden offspring circles are bolded to highlight the overlap in their core communities. Values to the right indicate the percent of reads represented by core microbes shared between common garden toads and wild toads at their ancestral sites (average ± SD). P-values from Wilcoxon signed rank test comparing proportion of reads in the overlap in ASVs versus OTUs. Relocated parent toads were excluded from Venn diagrams due to their small sample sizes.

Biology Open • Supplementary information

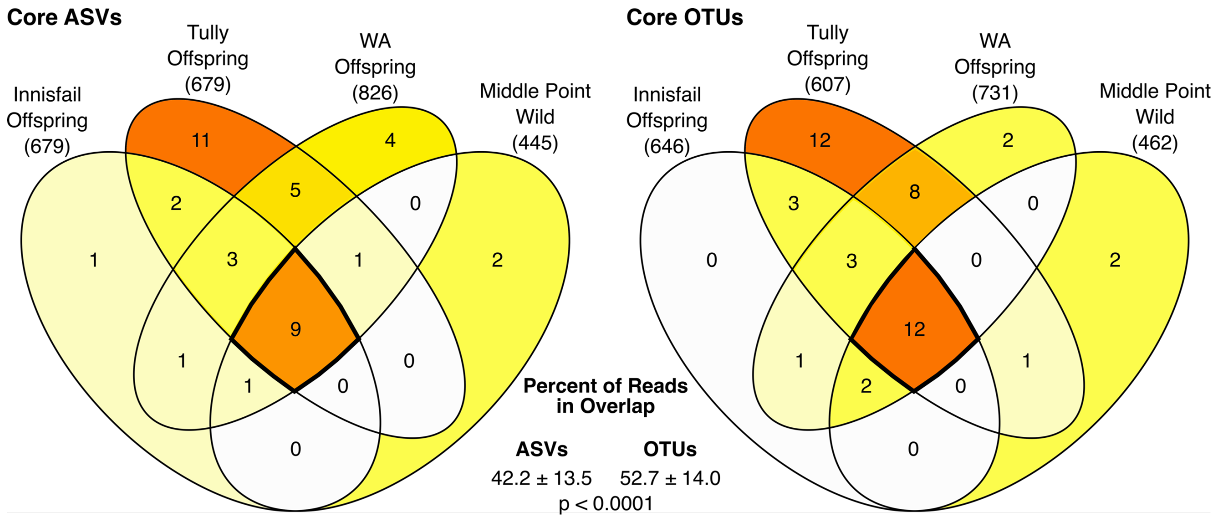

Fig. S6. Overlap of ASVs and OTUs in the core communities of common garden toads and nearby wild toads at Middle Point. Numbers and shading in the diagrams indicate number of microbes in the segment. P-value from Wilcoxon signed rank test comparing proportion of reads in the overlap in ASVs versus OTUs.

Biology Open • Supplementary information

Table S1. Results of pairwise contrasts of diversity of bacterial ASVs on cane toad skin in Australia. Pairwise comparisons are grouped by study question, among the nine groups of toads defined in Table 1. Asterisks indicate significant differences based on adjusted p-values < 0.05 (Tukey method for alpha diversities, Benjamini–Hochberg method for beta diversities), while underlined text represents instances where the result flipped significance in the OTU comparisons. Inn = Innisfail in Queensland. Tu = Tully in Queensland. MP = Middle Point in the Northern Territory. WA = toads captured near the invasion front in Western Australia. cap = individuals brought into captivity from the wild. off = offspring in common garden experiment. **Bold** in beta diversity comparisons indicate significant pairwise differences in dispersion. All global analyses of dispersion were significant. Note that Shannon diversity and Pielou's evenness for ASV data did not have a significant interaction between origin site and captivity, so we do not report pairwise contrasts for the ASV data of those metrics.

**Response**	**Wild Comparisons**		**Relocation & Parentage Comparisons**		**Common Garden Comparisons**		
			INNISFAIL	TULLY	AMONG OFFSPRING	VERSUS ANCESTRAL SITES	VERSUS NEARBY MP
Richness	Inn–Tu<br>Inn–MP<br>Inn–WA	Tu–MP<br>* Tu–WA<br>MP–WA	* Inn.cap–Inn<br>Inn.cap–Inn.off<br>* Inn.cap–MP	Tu.cap–Tu<br>Tu.cap–Tu.off<br>Tu.cap–MP	Inn.off–Tu.off<br>Inn.off–WA.off<br>Tu.off–WA.off	Inn.off–Inn<br>* Tu.off–Tu<br>WA.off– WA	* Inn.off–MP<br>Tu.off–MP<br>* WA.off–MP
Shannon	Inn–Tu<br>Inn–MP<br>Inn–WA	Tu–MP<br>Tu–WA<br>MP–WA	Inn.cap–Inn<br>Inn.cap–Inn.off<br>Inn.cap–MP	Tu.cap–Tu<br>Tu.cap–Tu.off<br>Tu.cap–MP	Inn.off–Tu.off<br>Inn.off–WA.off<br>Tu.off–WA.off	Inn.off–Inn<br>Tu.off–Tu<br>WA.off– WA	Inn.off–MP<br>Tu.off–MP<br>WA.off–MP
Faith's PD	Inn–Tu<br>Inn–MP<br>Inn–WA	Tu–MP<br>Tu–WA<br>MP–WA	* Inn.cap–Inn<br>Inn.cap–Inn.off<br>* Inn.cap–MP	Tu.cap–Tu<br>Tu.cap–Tu.off<br>Tu.cap–MP	Inn.off–Tu.off<br>Inn.off–WA.off<br>Tu.off–WA.off	* Inn.off–Inn<br>* Tu.off–Tu<br>WA.off– WA	* Inn.off–MP<br>Tu.off–MP<br>* WA.off–MP
Evenness	Inn–Tu<br>Inn–MP<br>Inn–WA	Tu–MP<br>Tu–WA<br>MP–WA	Inn.cap–Inn<br>Inn.cap–Inn.off<br>Inn.cap–MP	Tu.cap–Tu<br>Tu.cap–Tu.off<br>Tu.cap–MP	Inn.off–Tu.off<br>Inn.off–WA.off<br>Tu.off–WA.off	Inn.off–Inn<br>Tu.off–Tu<br>WA.off– WA	Inn.off–MP<br>Tu.off–MP<br>WA.off–MP
Jaccard	* **Inn–Tu**<br>* Inn–MP<br>* **Inn–WA**	* **Tu–MP**<br>* **Tu–WA**<br>* MP–WA	* **Inn.cap–Inn**<br>* **Inn.cap–Inn.off**<br>* **Inn.cap–MP**	* Tu.cap–Tu<br>* Tu.cap–Tu.off<br>* **Tu.cap–MP**	Inn.off–Tu.off<br>* Inn.off–WA.off<br>Tu.off–WA.off	* Inn.off–Inn<br>* **Tu.off–Tu**<br>* WA.off– WA	* Inn.off–MP<br>* Tu.off–MP<br>* WA.off–MP
Bray–Curtis	* **Inn–Tu**<br>* **Inn–MP**<br>* **Inn–WA**	* Tu–MP<br>* Tu–WA<br>* MP–WA	* **Inn.cap–Inn**<br>* **Inn.cap–Inn.off**<br>* Inn.cap–MP	* Tu.cap–Tu<br>* **Tu.cap–Tu.off**<br>* Tu.cap–MP	* Inn.off–Tu.off<br>* Inn.off–WA.off<br>* Tu.off–WA.off	* **Inn.off–Inn**<br>* Tu.off–Tu<br>* WA.off– WA	* Inn.off–MP<br>* Tu.off–MP<br>* WA.off–MP
Unweighted UniFrac	* Inn–Tu<br>* Inn–MP<br>* Inn–WA	* Tu–MP<br>* **Tu–WA**<br>* MP–WA	* **Inn.cap–Inn**<br>* **Inn.cap–Inn.off**<br>* **Inn.cap–MP**	* Tu.cap–Tu<br>* **Tu.cap–Tu.off**<br>* **Tu.cap–MP**	**Inn.off–Tu.off**<br>Inn.off–WA.off<br>Tu.off–WA.off	* Inn.off–Inn<br>* Tu.off–Tu<br>* WA.off– WA	* Inn.off–MP<br>* Tu.off–MP<br>* WA.off–MP
Weighted UniFrac	**Inn–Tu**<br>* **Inn–MP**<br>* **Inn–WA**	* Tu–MP<br>* Tu–WA<br>* MP–WA	* **Inn.cap–Inn**<br>* Inn.cap–Inn.off<br>* Inn.cap–MP	* Tu.cap–Tu<br>Tu.cap–Tu.off<br>* Tu.cap–MP	Inn.off–Tu.off<br>Inn.off–WA.off<br>Tu.off–WA.off	* **Inn.off–Inn**<br>* Tu.off–Tu<br>* WA.off– WA	* Inn.off–MP<br>* Tu.off–MP<br>* WA.off–MP

Biology Open • Supplementary information
